# Performance pressure and mental health among finance workers in Korea: a cross-sectional study

**DOI:** 10.4178/epih.e2023099

**Published:** 2023-11-07

**Authors:** Yu Min Lee, Hyoung-Ryoul Kim

**Affiliations:** 1 Department of Occupational and Environmental Medicine, Severance Hospital, Yonsei University College of Medicine, Seoul, Korea; 2 Department of Occupational and Environmental Medicine, Seoul St. Mary’s Hospital, College of Medicine, The Catholic University of Korea, Seoul, Korea

**Keywords:** Working group, Mental health, Finance worker, Suicide, Depression, Anxiety

## Abstract

**OBJECTIVES:**

While issues related to mental health, including suicide, have been frequently reported among finance workers, no formal investigation has been undertaken. This study was conducted to analyze correlations between indicators of performance pressure, which is a characteristic of the finance sector, and mental health.

**METHODS:**

An online survey was administered to 1,181 participants. Brief questionnaires were employed to ask general questions about participant characteristics, work environment, and the presence of performance pressure, suicidal thoughts/plans/attempts, depression (indicated by the Patient Health Questionnaire-9), and anxiety (measured with the Generalized Anxiety Disorder-7). Frequency analysis, chi-square testing, and multivariate logistic regression analysis were performed.

**RESULTS:**

Of the 1,181 respondents, 797 (83.5%) reported feeling pressure to perform at work, while 252 (26.4%) admitted to occasional willingness to achieve results even through illegal activities. Multivariate logarithmic regression analysis was employed to examine the correlation between performance pressure and mental health. This analysis revealed that those expressing willingness to achieve results through illegal activities exhibited higher levels of suicidal ideation (adjusted odds ratio [aOR], 1.63; 99% confidence interval [CI], 1.04 to 2.55), plans (aOR, 1.75; 99% CI, 1.01 to 3.01), and attempts (aOR, 2.72; 99% CI, 1.06 to 6.98). Additionally, these individuals demonstrated higher levels of depression (aOR, 2.02; 99% CI, 1.34 to 3.06) and anxiety (aOR, 2.84; 99% CI, 1.81 to 4.46).

**CONCLUSIONS:**

Performance pressure is prevalent within the finance industry. This pressure serves as a major source of stress for employees and is evident in 3 representative indicators of mental health: suicide, depression, and anxiety.

## GRAPHICAL ABSTRACT

[Fig f2-epih-45-e2023099]

## INTRODUCTION

While a sustainable competitive advantage can be secured by an organization largely through basic technical skills, greater emphasis is often placed on the competence of organizational members. Competitiveness hinges on the enhanced capabilities of human resources, which is why many companies prioritize human resource management [1]. Performance-oriented human resource management, which operates based on performance outcomes, is intended to improve both individual job performance and overall organizational results through differentiated evaluation and compensation according to performance [2]. To this end, performance indicators have been devised as a tool for the objective assessment of work outcomes. However, the performance pressures that arise in the process of achieving these indicators often manifest as individual feelings and have been defined in various ways. For instance, performance pressure has been described as “an individual’s negative emotional response arising from the concern that current performance is insufficient to achieve expected goals” [3]. More recently, certain researchers have defined it as “a desperate effort to achieve a high level of performance because performance is linked to important outcomes,” while others have described it as “the pressure one feels when they believe they must achieve a performance indicator.” Evaluation-based performance pressure is often utilized as an organizational management tool that spurs individuals or organizations towards growth, making it a key factor in the workplace [4–6]. By fostering a competitive work environment to manage organizational performance and tying rewards to achievement, managers inadvertently encourage the placement of performance pressure on employees [7]. If intended and measured indicators align and are paired with appropriate judgment from active professionals, then measurement can aid in evaluating the performance of individuals and organizations. However, issues arise when such measures become the basis for rewards and punishments—that is, when metrics become the foundation for performance-based pay or grades [8]. Performance pressure can have detrimental effects. Prior studies have suggested that such pressure is a double-edged sword, offering both benefits and drawbacks for employees and organizations. As a strategy to achieve high performance, it involves evaluation and supervision, and it may indeed improve performance by leveraging performance pressure and holding individuals accountable for results. Under pressure, employees often perceive that failure to meet performance standards will lead to meaningful negative consequences, such as being removed from a project or losing a job. They also view themselves negatively for not meeting standards due to their perceived lack of effort or incompetence [4,5,9]. Particularly in the financial sector, tangible indicators such as product sales volumes and contract amounts are made explicit. Consequently, numerous performance indicators are evaluated in real time, and individuals in the financial sector are immersed in a so-called performance-oriented culture that prioritizes the achievement of expected goals. Like other companies, those in the financial sector manage human resources through systems such as compensation and dismissal based on performance, and the resulting “performance pressure” culture is considered both an essential characteristic of the financial industry and something that its participants must endure.

Additionally, much like those in the service sector, finance workers frequently interact with people, and their work is intimately tied to fluctuations in the assets of these individuals. This contributes to a high level of job strain. A prior study indicated that finance workers were at a greater risk of burnout than health professionals, a difference attributed to the competitive nature of the finance industry [10]. In comparison to those in high-risk professions, such as healthcare providers and firefighters, a study from Cyprus found that many finance workers were subjected to a variety of challenging work environments and displayed a markedly increased risk of developing stress and depression. The study highlighted that the work environment of finance workers is characterized by intense competition and high demands for achievement. This environment increases their susceptibility to feelings of isolation, which in turn makes them more prone to experiencing sadness, over-reactivity, and agitation. Furthermore, while finance workers frequently express concerns about psychological stress, a strong tendency exists to focus solely on individual coping strategies, such as counseling [11].

In the current work culture and environment, frequent media reports have been published regarding mental health issues, including suicide, among financial workers. However, minimal research has been conducted on this topic. Consequently, the present study was undertaken to elucidate the actual working conditions and mental health of these workers, as well as to identify common causes of mental health issues across the organization. Additionally, this report examined the correlation between performance pressure indicators in office finance and mental health.

## MATERIALS AND METHODS

### Study participants

A cross-sectional study investigating the mental health of office finance workers was conducted through an online questionnaire from July 2, 2020 to August 31, 2020. The study targeted members of an office-based financial service union across the Korea. The National Office and Financial Services Workers’ Union, which encompasses most full-time office and financial service workers nationwide, represents approximately 50% of all employees in the Korean financial sector. To maximize the representativeness of the study group, union members were given equal opportunity to participate in the survey, regardless of their work area, sex, or age. The study included a total of 1,181 participants, and data on covariates were collected from the questionnaires. We considered the following variables as potential confounders: sex, age, income satisfaction, work type, and job category.

### Mental health-related occupational information

Prior to the survey, a focus group interview was conducted to gather information on factors intensifying the perceived workload. The questionnaire, targeted at members of the finance industry, then incorporated these factors. Considering the cultural characteristics of office finance workers who are obliged to meet key performance indicators, questions about performance pressure were formulated. These included: “I experience pressure to produce results in my work” (experience of performance pressure) and “At times, I have considered unethical actions to achieve results” (cheating for performance).

### Mental health outcomes

The Patient Health Questionnaire-9 (PHQ-9) is a shortened version of the PHQ, featuring 9 questions specifically designed to identify major depressive disorders [12]. Each question is evaluated using a 4-point Likert scale, ranging from 0 (not at all) to 3 (nearly every day). The severity of symptoms can be determined by the total score, with PHQ-9 scores of 5 to 9 indicating mild depressive symptoms, 10 to 14 suggesting moderate symptoms, 15 to 19 pointing to moderately severe symptoms, and 20 or above signifying severe depressive symptoms. The PHQ-9 has demonstrated validity through associations with anxiety, depressive cognitions, and discrimination [13].

The Generalized Anxiety Disorder-7 (GAD-7) is a tool composed of 7 questions, specifically designed to identify the presence of generalized anxiety disorders [14]. Individuals rate the frequency of 7 anxiety symptoms on a 4-point Likert-scale, ranging from 0 (not at all) to 3 (nearly every day). The severity of symptoms is determined through the total score; GAD-7 scores within the range of 5 to 9 suggest mild anxiety symptoms, scores of 10 to 14 indicate moderate anxiety symptoms, and scores of 15 or above denote severe anxiety symptoms. The GAD-7 has displayed validity through associations with depression, well-being, self-esteem, and discrimination [13].

To evaluate candidates for suicidal ideation, planning, and attempts, participants were asked to respond to the following questions concerning suicide: “Have you ever contemplated suicide?” “Have you ever devised a plan to commit suicide?” and “Have you ever made an attempt to commit suicide?”

### Statistical analysis

Maternal demographics and infant characteristics were presented using descriptive statistics, expressed as number (percentage), mean, and standard deviation. Regarding the factors intensifying workload, the 3 strongest factors were identified, and their frequencies were graphed. We employed logistic regression analysis to investigate the impact of performance pressure on mental health outcomes. To select covariates for inclusion in the adjusted models, we conducted a literature review to identify the risk factors associated with mental health outcomes. All statistical analyses were executed using SAS version 9.4 (SAS Institute Inc., Cary, NC, USA).

### Ethics statement

The Institutional Review Board of Chung-Ang University approved this study (1041078-201712-HRSB-239-01C), and written informed consent was obtained from each participant.

## RESULTS

**Figure 1:**
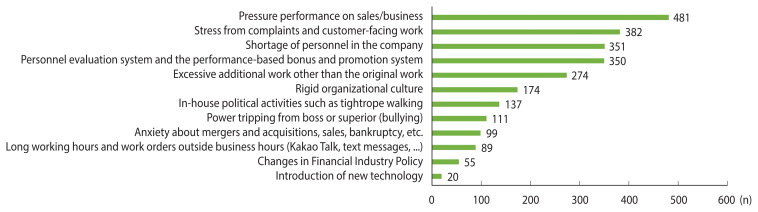
Factors perceived as intensifying the workload of finance workers.

Table 1 outlines the characteristics of the participants. Of the 1,181 respondents, 60.5% were male, and the median age was 41 years. Regarding job tenure, the largest group of participants (39.4%) had been in their roles for at least 10 years but less than 20 years. Since occupational characteristics vary based on work type and job category, the distribution of each was verified. Work was broadly categorized into 4 types, with insurance being the most common, accounting for 39.0%. Jobs were divided into 6 categories, with “management and support, main office” being the most frequent at 36.4%. When participants were asked to identify factors increasing the labor demands on financial workers, with multiple responses allowed, performance pressure was the most frequently selected (Figure 1).

Of the respondents, 797 individuals (83.5%) reported feeling pressure to perform at work, while 252 (26.4%) admitted to at least occasional willingness to achieve results, even if it meant engaging in illegal activities (Table 2). Notably, over 40% of those in the sales field, both at the main and branch levels, reported experiencing extreme performance pressure. Each mental health outcome was more frequently reported among females. In terms of work type, those in the insurance sector reported the highest frequencies of anxiety (47.5%), depression (30.4%), and suicide attempts (6.0%). When classified by job category, workers in “sales, branch office” (52.2%) and “call center” roles (48.3%) reported high levels of anxiety. Furthermore, those in “management and support, branch office” roles reported an exceptionally high rate of suicide attempts (7.0%).

Regarding the correlation between performance pressure and mental health, univariate and multivariate logistic regression analysis revealed that anxiety (aOR, 2.39; 99% confidence interval [CI], 1.36 to 4.21) was higher among those who perceived pressure to perform at work compared to those who did not. Individuals who felt compelled to achieve results, even if it meant engaging in illegal activities, exhibited higher rates of suicidal ideation (aOR, 1.63; 99% CI, 1.04 to 2.55), plans (aOR, 1.75; 99% CI, 1.01 to 3.01), and attempts (aOR, 2.72; 99% CI, 1.06 to 6.98). These individuals also demonstrated higher levels of depression (aOR, 2.02; 99% CI, 1.34 to 3.06) and anxiety (aOR, 2.84; 99% CI, 1.81 to 4.46) (Table 3).

When the data were stratified based on work type, each group reported consistent results (Table 4). Similarly, when stratified by job category, each mental health outcome was found to be statistically significantly higher in both the “management and support, main office” and “management and support, branch office” groups, particularly in response to questions about performance pressure (Supplementary Material 1).

## DISCUSSION

We examined the impact of performance pressure on mental health issues, including anxiety, depression, and suicidal tendencies, among finance workers. The pressure to meet performance indicators and the challenges faced when dealing with customers were identified as factors intensifying the workload in the finance sector. Individuals subjected to performance pressure were found to have an elevated risk of developing anxiety. Notably, performance pressure was found to amplify the risk of anxiety in males by more than 3-fold. Furthermore, those who contemplated engaging in illegal activities due to such pressure were observed to have an increased risk of experiencing anxiety, depression, suicidal ideation, suicide plans, and suicide attempts.

In 2021, the lifetime prevalence rates of anxiety and depressive disorders among Koreans were reported to be 9.3% and 7.7%, respectively, while the respective incidences of suicidal thoughts, plans, and attempts stood at 10.7%, 2.5%, and 1.7%. However, the frequency of mental health disorders among the participants in this study was notably higher, with respective rates of 42.2%, 28.6%, 32.7%, 16.3%, and 4.4% reported. The 2021 study also indicated a high prevalence of mental illness in female, a trend that was consistently observed in our research as well [15]. Interestingly, despite the high incidence of anxiety in female, the risk of anxiety according to performance indicators was found to be higher in male. This is believed to be due to sex-based differences in risk factors for anxiety. In general, factors influencing anxiety can be categorized into socio-demographic, psychosocial, and health status (both mental and physical) factors. More specifically, these include sex, childhood experiences, financial difficulties, physical health challenges, and stress [16]. For male, social determinants are emphasized, and anxiety can arise when they are unable to fulfill the role of a so-called typical man due to socially defined masculine roles and values [17]. Traditionally, male have been expected to assume social roles more actively than female, and as a result, male’s status has been primarily evaluated based on the outcomes of their social roles and activities. This is particularly relevant in Korean culture, where male are expected to endure occupational difficulties as family heads. In such situations, male tend to place more importance on socio-cultural and socioeconomic factors than female do, and they react with greater sensitivity to these factors [18,19]. This trend has been similarly observed in cases of suicide and suicide attempts [20,21]. Therefore, the high reported risk can be concluded to stem from the pressure to perform, which threatens male’s social role.

Previous research has demonstrated that employees subjected to performance pressure often experience heightened anxiety, diminished attention spans, and ultimately, a decline in performance [22–24]. Such pressures can also undermine fundamental needs, such as the need to showcase one’s capabilities [25]. Failure to meet these demands can result in a self-perception of incompetence, leading in turn to extended working hours. In extreme cases, if performance becomes the determinant of future employment status, it can endanger objectives like job security and stable income. This situation can induce anger over the existence of performance pressure, potentially leading to aggressive behavior towards others as a means to alleviate this anger [26]. Anger is a self-protective emotion that emerges when the ego perceives a threat. People experience anger at work in 3 primary contexts: goal intervention, perception of inequity, and interpersonal conflict. Consequently, when situations arise in the workplace that hinder individuals from achieving their goals, anger is particularly likely to result. Performance pressures repeatedly expose employees to these situations [27].

The financial workforce is subject to rapid changes, influenced by both the financial climate of individual countries and the global financial situation. This constant flux often presents challenges in acquiring new skills. During financial crises, job instability within this sector tends to increase. Organizations often respond to these crises through restructuring, which can lead to increased workloads and longer working hours, more temporary contracts, and further job instability. Additionally, the real-time generation of knowledge and information required by these organizations can create pressure to process information quickly and simultaneously. As the work environment becomes increasingly flexible, without time and space constraints, managing workload can become challenging [28,29]. According to a United Kingdom report, the finance industry topped the list of the 10 industries most likely to involve work-related stress. In 2020, the telecommunications sector was separated from this list and ranked second, leaving the finance industry, excluding telecommunications, in eighth place. This indicates that the finance industry harbors substantial work stress, with the telecommunications sector identified as a particularly high-risk group [30]. Even within the area of finance, work environments vary greatly depending on the type of work or job category. Factors such as the use of qualitative or quantitative indicators as performance measures, the use of face-to-face work, and the support system within an organization can contribute to individual psychological burdens. For example, in the “loans, savings, and deposits” sector, compensation is directly linked to performance, which can intensify performance pressure. Among job categories, those in “management and support” roles often deal directly with customers at the forefront of the organization, thus experiencing emotional labor. This vital role involves mediating between the organization and its customers in a manner that minimizes friction, which can result in substantial psychological stress.

Performance indicators within an organization are relative and subject to change based on the organization’s current state. Consequently, in certain instances, inappropriate performance indicators may be chosen. Often, the process of selection or evaluation of these indicators is not transparent, which can contribute to a sense of organizational injustice. In many situations, performance indicators are not meticulously chosen but are merely presented as quotas for a shared objective. These quotas are then used as tools to manage employees, with the responsibility often shifted onto individuals. Furthermore, when results fall short of expectations, they are frequently personalized as a deficiency in the worker’s competency, leading to perceived inferiority within the organization. This hinders questioning of the evaluation system. Campbell’s law [31], which discusses the performance paradox related to goal quantification, is also known as measure fixation. This law suggests that the more quantitative measurement indicators are used in performance evaluation, the higher the likelihood of distortion. This highlights the risks associated with the quantification of indicators and suggests that the greater the reliance on quantitative indicators, the more susceptible these indicators become to corruption pressures. In numerous instances, an employee may not be able to resolve issues that have arisen, leading to a tendency to alleviate stress through drinking or smoking. Mental health issues are particularly concerning, as they can impact overall health levels. Specifically, the more fragmented the performance index, the more individuals perceive unsolvable problems as personal failures, which can obstruct opportunities for organizational improvement. Given that an organization’s management goal is typically its integrated growth, considerable effort should be invested in selecting performance indicators. Additionally, systematic support, such as appropriate work adjustment, relocation, and education, is necessary for individuals with low specific performance. This need has been explored in numerous studies under the concept of job crafting, with many studies focusing on specific realization methods [32–35]. Job crafting can lead to physical and cognitive changes in one’s task and relational domain [36]. Through this process, workers can find meaning and identity in their work, and underperformers can align their experiences and abilities with the organization’s goals. Therefore, it is essential for all members of an organization, including underperformers, to collaborate in achieving both individual and organizational goals or satisfaction.

This study does present certain limitations. Given its cross-sectional nature, it is challenging to infer a causal relationship due to the inability to specify temporal relations. Furthermore, the representation of the entire population may be limited, as this survey was conducted solely among regular employees in the finance sector. Despite these limitations, this research offers value as the inaugural study on the mental health of financial workers in Korea, and it medically investigated the performance pressure inherent in the Korean financial industry’s work environment. Prior to survey construction, focus group interviews were conducted and the results were used to develop the core questionnaire, contributing qualitative value. Consequently, this is the only study that can independently verify that different occupations within the financial industry have varying impacts on health.

In conclusion, performance pressure is recognized as a characteristic of employment within the finance industry. This pressure serves as a substantial source of stress for employees and manifests in 3 key indicators of mental health: suicide, depression, and anxiety. Similar to how performance indicators require a systematic approach, addressing the issue of performance pressure also necessitates a systematic solution.

## Figures and Tables

**Figure f2-epih-45-e2023099:**
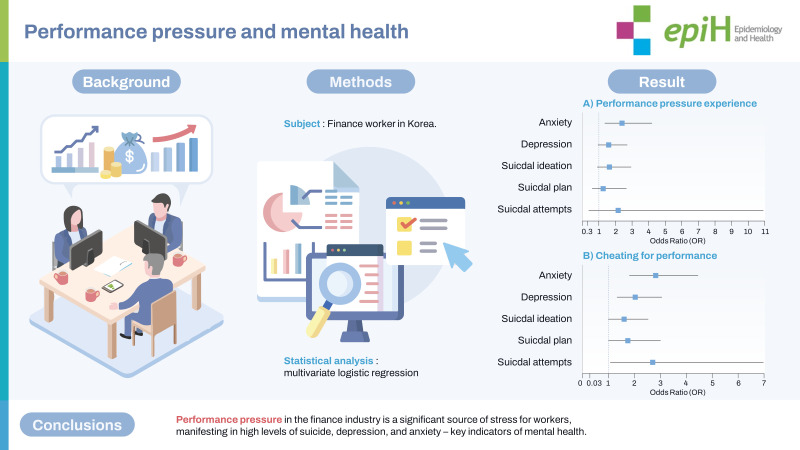


**Table 1.** Participant characteristics

Characteristics	n (%) or median [IQR]
Total (n)	1,181
Sex	
Male	714 (60.5)
Female	467 (39.5)
Age (yr)	41 [35, 46]
20 to <30	94 (8.0)
30 to <40	430 (36.6)
40 to <50	520 (44.2)
50 to <60	129 (11.0)
≥60	3 (0.3)
Missing data	5
Total income in last year (10,000 KRW)	7,000 [5,000-10,000]
Missing data	211
Job tenure (yr)	
<5	163 (13.9)
5 to <10	247 (21.1)
10 to <20	461 (39.4)
≥20	299 (25.6)
Missing data	11
Work type	
Loans, savings, and deposits	353 (29.9)
Insurance	461 (39.0)
Securities	225 (19.0)
Others^1^	142 (12.0)
Job category	
Management and support, main office	421 (36.4)
Management and support, branch office	276 (23.8)
Sales, main office	49 (4.2)
Sales, branch office	274 (23.7)
Call center	40 (3.5)
IT and computer systems	97 (8.4)
Missing data	24

IQR, interquartile range; KRW, Korean won; IT, information technology.

1 Included the Korea Deposit Insurance Corporation, Public Officials Benefit Association, cooperative unions, etc.

**Table 2.** Performance pressure and mental health outcomes in finance workers

Variables	(A) Experience of performance pressure^1^	(B) Cheating for performance^1^	Anxiety	Depression	Suicidal ideation	Suicide plan	Suicide attempt
Total	797 (83.5)	252 (26.4)	359 (42.2)	338 (28.6)	277 (32.7)	138 (16.3)	37 (4.4)
Sex							
Male	516 (87.6)	189 (32.1)	211 (40.4)	199 (27.9)	162 (31.1)	81 (15.6)	19 (3.6)
Female	281 (77.0)	63 (17.3)	148 (45.1)	139 (29.8)	115 (35.2)	57 (17.4)	18 (5.5)
Work type							
Loans, savings, and deposits	238 (84.4)	70 (24.8)	97 (38.0)	97 (27.5)	90 (35.4)	37 (14.6)	8 (3.1)
Insurance	308 (84.6)	97 (26.6)	152 (47.5)	140 (30.4)	99 (31.1)	52 (16.3)	19 (6.0)
Securities	153 (78.1)	60 (30.6)	67 (38.5)	63 (28.0)	52 (29.9)	31 (17.8)	2 (1.1)
Others	98 (87.5)	25 (22.3)	43 (42.6)	38 (26.8)	36 (35.6)	18 (17.8)	8 (7.9)
Job category							
Management and support, main office	276 (78.4)	65 (18.5)	129 (40.3)	131 (31.1)	102 (32.0)	43 (13.5)	13 (4.1)
Management and support, branch office	178 (80.9)	58 (26.4)	80 (40.2)	80 (29.0)	74 (37.2)	41 (20.6)	14 (7.0)
Sales, main office	38 (95.0)	16 (40.0)	13 (41.9)	12 (24.5)	10 (33.3)	3 (10.0)	0 (0.0)
Sales, branch office	224 (94.9)	103 (43.6)	106 (52.2)	83 (30.3)	65 (32.2)	40 (19.8)	9 (4.5)
Call center	26 (83.9)	4 (12.9)	14 (48.3)	13 (32.5)	10 (34.5)	6 (20.7)	1 (3.4)
IT and computer systems	54 (73.0)	6 (8.1)	17 (25.4)	19 (19.6)	16 (23.9)	5 (7.5)	0 (0.0)

Values are presented as number (%). IT, information technology.

1 Responses to the following questions: (A) “I experience pressure to produce results in my work” and (B) “At times, I have considered unethical actions to achieve results”.

**Table 3.** Association between performance pressure and mental health outcomes

Variables	n	OR (99% CI)	n	aOR (99% CI)^1^
(A) Experience of performance pressure^2^				
All participants				
Anxiety	850	2.20 (1.29, 3.74)	846	2.39 (1.36, 4.21)
Depression	954	1.50 (0.92, 2.46)	950	1.60 (0.94, 2.71)
Suicidal ideation	847	1.63 (0.94, 2.84)	843	1.63 (0.91, 2.93)
Suicide plan	847	1.44 (0.70, 2.94)	843	1.25 (0.60, 2.64)
Suicide attempt	847	2.23 (0.46, 10.71)	843	2.16 (0.43, 10.91)
Male				
Anxiety	522	2.70 (1.19, 6.10)	521	3.16 (1.36, 7.33)
Depression	589	1.52 (0.74, 3.15)	588	1.74 (0.82, 3.72)
Suicidal ideation	520	1.41 (0.64, 3.10)	519	1.41 (0.63, 3.16)
Suicide plan	520	1.58 (0.53, 4.66)	519	1.50 (0.49, 4.53)
Suicide attempt	520	1.20 (0.17, 8.50)	519	1.20 (0.16, 8.87)
Female				
Anxiety	328	2.03 (0.98, 4.20)	325	1.99 (0.90, 4.41)
Depression	365	1.61 (0.81, 3.21)	362	1.46 (0.69, 3.10)
Suicidal ideation	327	2.02 (0.92, 4.44)	324	1.98 (0.85, 4.63)
Suicide plan	327	1.40 (0.53, 3.70)	324	1.12 (0.40, 3.14)
Suicide attempt	327	5.07 (0.35, 73.46)	324	4.10 (0.25, 66.19)
(B) Cheating for performance^2^				
All participants				
Anxiety	850	2.52 (1.66, 3.84)	846	2.84 (1.81, 4.46)
Depression	954	1.84 (1.25, 2.72)	950	2.02 (1.34, 3.06)
Suicidal ideation	847	1.61 (1.05, 2.46)	843	1.63 (1.04, 2.55)
Suicide plan	847	1.76 (1.05, 2.95)	843	1.75 (1.01, 3.01)
Suicide attempt	847	2.45 (1.02, 5.92)	843	2.72 (1.06, 6.98)
Male				
Anxiety	522	2.82 (1.69, 4.70)	521	3.22 (1.85, 5.58)
Depression	589	1.85 (1.16, 2.97)	588	1.96 (1.19, 3.22)
Suicidal ideation	520	1.55 (0.92, 2.62)	519	1.49 (0.87, 2.58)
Suicide plan	520	1.47 (0.76, 2.83)	519	1.30 (0.65, 2.59)
Suicide attempt	520	2.87 (0.85, 9.62)	519	2.71 (0.76, 9.60)
Female				
Anxiety	328	2.31 (1.07, 4.97)	325	2.38 (1.04, 5.41)
Depression	365	2.20 (1.07, 4.53)	362	2.28 (1.06, 4.91)
Suicidal ideation	327	1.95 (0.92, 4.16)	324	2.04 (0.92, 4.55)
Suicide plan	327	2.65 (1.13, 6.25)	324	2.93 (1.19, 7.19)
Suicide attempt	327	2.47 (0.64, 9.50)	324	2.43 (0.58, 10.20)

OR, odds ratio; CI, confidence interval; aOR, adjusted odds ratio.

1 Adjusted for sex, age group, job tenure, and total income in the last year.

2 Responses to the following questions: (A) “I experience pressure to produce results in my work” and (B) “At times, I have considered unethical actions to achieve results”.

**Table 4.** Associations between performance pressure and mental health outcomes by work type (multivariate)^1^

Variables	n	Loans, savings, and deposits	n	Insurance	n	Securities	n	Others
(A) Experience of performance pressure^2^								
Anxiety	255	3.16 (0.99, 10.09)	319	1.87 (0.73, 4.82)	171	3.21 (0.95, 10.84)	101	1.19 (0.19, 7.30)
Depression	282	1.59 (0.59, 4.28)	363	2.10 (0.84, 5.21)	193	1.80 (0.55, 5.87)	112	0.62 (0.13, 3.09)
Suicidal ideation	254	3.29 (0.93, 11.67)	317	0.84 (0.32, 2.17)	171	1.96 (0.58, 6.60)	101	1.53 (0.22, 10.70)
Suicide plan	254	2.10 (0.39, 11.30)	317	0.80 (0.25, 2.57)	171	2.17 (0.44, 10.83)	101	0.91 (0.09, 8.99)
Suicide attempt	-	-	317	1.53 (0.18, 13.19)	-	-	101	0.63 (0.02, 20.23)
(B) Cheating for performance^2^								
Anxiety	255	2.30 (0.95, 5.55)	319	2.79 (1.32, 5.90)	171	2.83 (1.05, 7.59)	101	7.28 (1.43, 36.92)
Depression	282	2.33 (1.01, 5.34)	363	1.86 (0.97, 3.58)	193	2.07 (0.80, 5.38)	112	2.77 (0.73, 10.50)
Suicidal ideation	254	1.58 (0.67, 3.72)	317	1.74 (0.83, 3.66)	171	1.71 (0.64, 4.57)	101	1.53 (0.34, 6.77)
Suicide plan	254	2.84 (0.99, 8.16)	317	1.47 (0.60, 3.61)	171	1.60 (0.49, 5.22)	101	0.96 (0.14, 6.48)
Suicide attempt	254	3.18 (0.40, 25.20)	317	2.57 (0.66, 9.96)	171	1.63 (0.04, 68.98)	101	1.82 (0.12, 28.04)

Values are presented as adjusted odds ratio (99% confidence interval).

1 Adjusted for sex, age group, job tenure, and total income in the last year.

2 Responses to the following questions: (A) “I experience pressure to produce results in my work” and (B) “At times, I have considered unethical actions to achieve results”.
