# Supplementary material for: Performance pressure and mental health among finance workers in Korea: a cross-sectional study
Source: Epidemiol Health. 2023 Nov 7;45:e2023099. doi: 10.4178/epih.e2023099 (PMC10876446; doi:10.4178/epih.e2023099)
Supplement: Supplementary file 1 [file epih-45-e2023099-Supplementary.docx]

Supplementary Materials 1. Associations between performance pressure and mental health outcomes by job categories (multivariate).

| (A) Performance pressure experience | | | | | | | | |
| --- | --- | --- | --- | --- | --- | --- | --- | --- |
|  | Managing and supporting for main office | | | | Managing and supporting for branch office | | | |
|  | N | aOR | 99%CI | | N | aOR | 99%CI | |
| Anxiety | 310 | 1.56 | 0.70 | 3.48 | 193 | 3.00 | 0.84 | 10.68 |
| Depression | 342 | 1.36 | 0.63 | 2.90 | 213 | 2.82 | 0.87 | 9.16 |
| Suicidal ideation | 309 | 2.10 | 0.85 | 5.16 | 193 | 1.07 | 0.34 | 3.37 |
| Suicidal plan | 309 | 1.75 | 0.50 | 6.16 | 193 | 1.08 | 0.26 | 4.42 |
| Suicidal attempts | 309 | 1.82 | 0.22 | 14.89 | 193 | 2.69 | 0.15 | 49.61 |
| (B) Cheating for performance | | | | | | | | |
|  | Managing and supporting for main office | | | | Managing and supporting for branch office | | | |
|  | N | aOR | 99%CI | | N | aOR | 99%CI | |
| Anxiety | 310 | 2.48 | 1.11 | 5.57 | 193 | 4.22 | 1.53 | 11.59 |
| Depression | 342 | 2.20 | 1.03 | 4.68 | 213 | 2.65 | 1.06 | 6.61 |
| Suicidal ideation | 309 | 2.30 | 1.03 | 5.11 | 193 | 2.80 | 1.07 | 7.33 |
| Suicidal plan | 309 | 3.08 | 1.13 | 8.35 | 193 | 2.89 | 1.00 | 8.34 |
| Suicidal attempts | 309 | 2.51 | 0.47 | 13.44 | 193 | 4.75 | 0.86 | 26.09 |
| ^a^ Adjusted sex, age group, job tenure, total income in last year.  aOR ; adjusted Odds Ratio | | | | | | | | |

Supplementary Materials 1. Associations between performance pressure and mental health outcomes by job categories (continued).

| (A) Performance pressure experience | | | | | | | | | | | | | | | | |
| --- | --- | --- | --- | --- | --- | --- | --- | --- | --- | --- | --- | --- | --- | --- | --- | --- |
|  | Sales in main office | | | | Sales in branch office | | | | Call center | | | | IT and computer system | | | |
|  | N | aOR | 99%CI | | N | aOR | 99%CI | | N | aOR | 99%CI | | N | aOR | 99%CI | |
| Anxiety | - | - | - | - | 200 | 4.45 | 0.52 | 38.35 | 29 | 16.04 |  |  | 63 | 2.04 | 0.24 | 17.59 |
| Depression | 39 | 0.66 | 0.01 | 36.99 | 232 | 2.98 | 0.36 | 24.92 | 31 | 4.57 | 0.11 | 198.4 | 69 | 1.83 | 0.30 | 11.12 |
| Suicidal ideation | 29 | 0.09 |  |  | 199 | 2.28 | 0.24 | 21.81 | 29 | 2.27 | 0.06 | 87.84 | 63 | 1.24 | 0.15 | 10.23 |
| Suicidal plan | 29 | - | - | - | 199 | 0.70 | 0.01 | 20.58 | 29 | 0.40 | 0.01 | 20.58 | 63 | 0.38 | 0.02 | 7.25 |
| Suicidal attempts |  |  |  |  | - | - | - | - | - | - | - | - | - | - | - | - |
| (B) Cheating for performance | | | | | | | | | | | | | | | | |
|  | Sales in main office | | | | Sales in branch office | | | | Call center | | | | IT and computer system | | | |
|  | N | aOR | 99%CI | | N | aOR | 99%CI | | N | aOR | 99%CI | | N | aOR | 99%CI | |
| Anxiety | 30 | 19.92 |  |  | 200 | 1.71 | 0.75 | 3.88 | 29 |  |  |  | 63 | 4.37 | 0.09 | 219.4 |
| Depression | 39 | 3.06 | 0.28 | 32.96 | 232 | 1.38 | 0.65 | 2.94 | 31 | 2.97 | 0.05 | 178.4 | 69 | 16.10 | 0.48 | 536.1 |
| Suicidal ideation | 29 | 0.67 | 0.04 | 11.39 | 199 | 0.86 | 0.37 | 2.00 | 29 | 3.27 | 0.05 | 226.6 | 63 | 0.63 | 0.02 | 21.65 |
| Suicidal plan | 29 | - | - | - | 199 | 0.73 | 0.26 | 2.00 | 29 | - | - | - | 63 | 1.99 | 0.04 | 108.0 |
| Suicidal attempts |  | - | - | - | 199 | 1.76 | 0.25 | 12.23 | 29 | - | - | - |  | - | - | - |
| ^a^ Adjusted sex, age group, job tenure, total income in last year.  aOR ; adjusted Odds Ratio | | | | | | | | | | | | | | | | |
